# *Vibrio coralliilyticus* infection triggers a behavioural response and perturbs nutritional exchange and tissue integrity in a symbiotic coral

**DOI:** 10.1038/s41396-018-0327-2

**Published:** 2018-12-12

**Authors:** E. Gibbin, A. Gavish, T. Krueger, E. Kramarsky-Winter, O. Shapiro, R. Guiet, L. Jensen, A. Vardi, A. Meibom

**Affiliations:** 1 Laboratory for Biological Geochemistry, School of Architecture, Civil and Environmental Engineering, École Polytechnique Fédérale de Lausanne (EPFL), Lausanne, Switzerland; 2 Department of Plant and Environmental Sciences, Weizmann Institute of Science, Rehovot, Israel; 3 Volcani Center for Agricultural Research, Rishon LeZion, Israel; 4 BioImaging and Optics Core Facility, École Polytechnique Fédérale de Lausanne (EPFL), Lausanne, Switzerland; 5 Center for Advanced Surface Analysis, Institute of Earth Sciences, University of Lausanne, Lausanne, Switzerland

**Keywords:** Microbial ecology, Water microbiology, Metabolism

## Abstract

Under homoeostatic conditions, the relationship between the coral *Pocillopora damicornis* and *Vibrio coralliilyticus* is commensal. An increase in temperature, or in the abundance of *V. coralliilyticus*, can turn this association pathogenic, causing tissue lysis, expulsion of the corals’ symbiotic algae (genus *Symbiodinium*), and eventually coral death. Using a combination of microfluidics, fluorescence microscopy, stable isotopes, electron microscopy and NanoSIMS isotopic imaging, we provide insights into the onset and progression of *V*. *coralliilyticus* infection in the daytime and at night, at the tissue and (sub-)cellular level. The objective of our study was to connect the macro-scale behavioural response of the coral to the micro-scale nutritional interactions that occur between the host and its symbiont. In the daytime, polyps enhanced their mucus production, and actively spewed pathogens. *Vibrio* infection primarily resulted in the formation of tissue lesions in the coenosarc. NanoSIMS analysis revealed infection reduced ^13^C-assimilation in *Symbiodinium*, but increased ^13^C-assimilation in the host. In the night incubations, no mucus spewing was observed, and a mucus film was formed on the coral surface. *Vibrio* inoculation and infection at night showed reduced ^13^C-turnover in *Symbiodinium*, but did not impact host ^13^C-turnover. Our results show that both the nutritional interactions that occur between the two symbiotic partners and the behavioural response of the host organism play key roles in determining the progression and severity of host-pathogen interactions. More generally, our approach provides a new means of studying interactions (ranging from behavioural to metabolic scales) between partners involved in complex holobiont systems, under both homoeostatic and pathogenic conditions.

## Introduction

Scleractinian corals host numerous microbial symbionts with different types of interactions [1]. One single, relatively large reef-building coral colony contains billions of dinoflagellate algae, bacteria, archaea, fungi, and viruses [2–5], that are collectively known as the coral holobiont [6]. The relationship between the coral animal and its endosymbiotic dinoflagellate population (genus *Symbiodinium*) has become emblematic of mutualistic symbiosis in the marine environment. *Symbiodinium* cells live intracellularly within coral cells and provide the coral animal with a reliable food source via translocation of photosynthates and their immediate derivatives (sugars, lipids, and amino acids [7–9]). Concurrently, corals host a diverse array of bacteria, both in a thin layer of mucus on the surface of the coral [10–14] and deeper within their tissues and skeleton [15].

Coral-dinoflagellate and coral-bacteria interactions are particularly sensitive to fluctuations in seawater temperature. Elevated temperature is known to perturb the allocation of carbon and nitrate between *Symbiodinium* and their host [16–18], but the mechanisms responsible for these perturbations are debated. Shifts in the coral microbiome also occur during periods of thermal stress [19]. One of the most consistent changes reported is a sharp increase in bacteria belonging to the genus *Vibrio*. This genus is present in the microbiome of healthy corals at low densities [20–22], but increases in abundance by several orders of magnitude under elevated temperature [23]. One species, *V. coralliilyticus*, is particularly responsive to these fluctuations [23, 24]. It is best known as the aetiological agent of bacterial-bleaching in *Pocillopora damicornis* [25–27], but is also present in other coral diseases, including “white syndrome” [28, 29] and black band disease [30]. Both the virulence [28] and antimicrobial resistance [31] of *V. coralliilyticus* are strongly correlated with temperature. Under high temperatures, *V. coralliilyticus* genes involved in motility, resistance, and host degradation are up-regulated [32], increasing the pathogens’ speed and accuracy of finding suitable coral targets [33]. This *Vibrio* uses dimethylsulfoniopropionate (DMSP), a compound produced by both the coral animal and its algal symbiont [34], to locate physiologically-stressed corals [35, 36]. Once contact is made, the pathogen enters the polyp via its mouth or through tears in the coral’s tissue [37]. Inside the polyp, *V. coralliilyticus* cells divide [27] and secrete enzymatic proteins that help them attach to- and penetrate deeper into the tissues. One group of proteins, matrix metalloproteases (MMPs), are thought to play a particularly important role in infection dynamics [26, 38, 39], by causing tissue lesion formation [38] and affecting the photosynthetic efficiency of the algal symbiont [40, 41].

It is still unclear how the in situ increase in pathogen abundance influences coral metabolism. In part, this is due to the complexity of quantifying the flux of metabolites between the three partners in the holobiont (the bacteria, the dinoflagellate and the host). Nanoscale secondary ion mass spectrometry (NanoSIMS) [42] combined with isotopically-labelled seawater (^13^C-enriched bicarbonate) permits the tracing of assimilated and translocated carbon between the algal symbionts and their host [9, 16, 43]. It also facilitates the visualization of isotopically-labelled pathogens and/or their degradation/secretion products within coral tissue [37, 44]. Here, we combine these two approaches in a first case study of the *P. damicornis-V. coralliilyticus* model. By using ^15^N-labelled bacteria and ^13^C-labelled seawater, we are able to determine if the presence of pathogens influences resource partitioning in the coral holobiont. Specifically, we quantify: (1) ^13^C-assimilation in *Symbiodinium* and the amount of ^13^C-labelled photosynthates that are assimilated by the host; (2) the metabolic turnover of ^13^C in *Symbiodinium* and in their host and (3) the incorporation of bacterial-derived ^15^N within the tissues of the coral holobiont.

## Methods

### Coral collection and pre-inoculation conditions

Two Red Sea *P. damicornis* colonies were collected at ~8 m depth from a coral nursery situated adjacent to the Inter-University Institute for Marine Sciences (Eilat, Israel). Small, apical branch tips (5 × 5 mm) were clipped from each colony and transferred to an outdoor aquarium, where they were supplied with ambient seawater (28.5 ± 0.5 °C) and natural, but shaded sunlight (300–400 μmol photons m^−2^ s^−1^ at 12:00). After one week, the fragments were transported to Weizmann Institute of Science (Rehovot, Israel) and placed in a custom-built raceway system containing 0.22 μm-filtered seawater (FSW) [45]. Temperature (28.0 ± 0.5 °C) was controlled using a 25 W aquarium heater (Aqua Medic), while light (150 μmol photons m^−2^ s^−1^, 13.5 h light: 10.5 h dark) was provided by blue and white LEDs. Distilled water was added to the system on a daily basis to compensate for any water lost through evaporation and to prevent fluctuations in salinity. Fragments were maintained in the set-up for a maximum of 3 weeks while experiments were conducted. Three days before the start of each experiment, ten fragments were selected at random and transferred to a second raceway system held under the same light regime but at elevated temperatures (31.0 ± 1.0 °C). Experimental fragments were selected after visual confirmation of health (i.e., skeleton completely covered by tissue, polyps extended, no paling of the coenosarc, and no excess mucus production).

### The Microfluidic Coral Infection (MCI) experimental platform

**Fig. 1:**
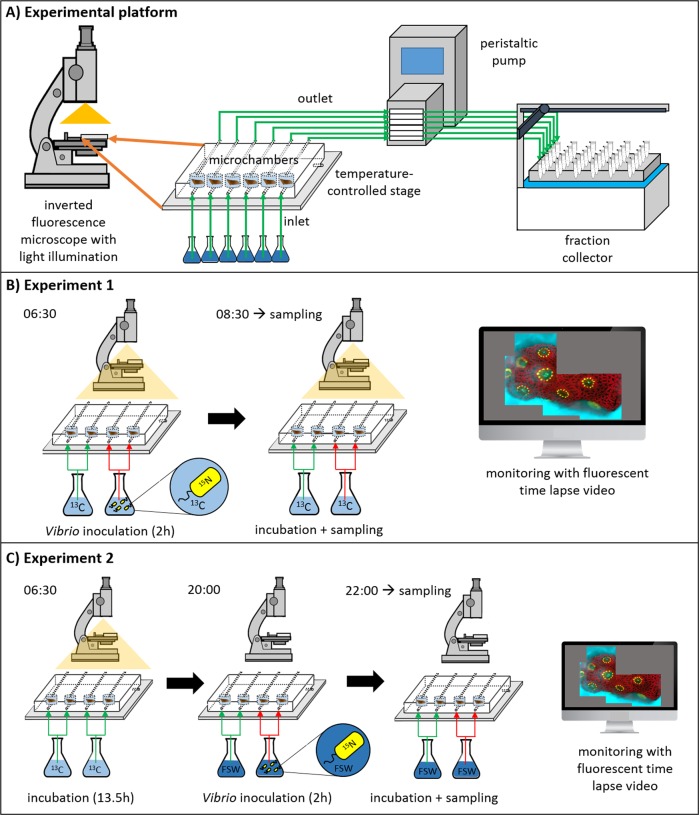
Schematic overview of the Microfluidic Coral Infection platform and the experimental design. **a** Overview of the elements of the microfluidic experimental platform used for investigating **b** the effect of *Vibrio coralliilyticus* infection on daytime coral carbon assimilation and **c** the effect of a night-time *Vibrio* infection on the metabolic turnover of the assimilated photo-autotrophic carbon from the previous light phase. Note the presence and absence of light illumination in different phases of the experiments. Specific sampling times for infected fragments (and their corresponding control fragments) were chosen according to the visual manifestation of signs of infection (see main text)

This system is described in detail in Gavish et al. [39], but we have provided a schematic of the experimental set-up (Fig. 1a). Briefly, coral fragments were placed in microchambers (Ø = 8 mm, depth = 5 mm), crafted from polydimethylsiloxane and plasma-bonded to a glass microscope slide. An ApopTag plastic cover slip (Merck) sealed the micro-chambers and the chip was mounted on a temperature-controlled stage of an inverted fluorescence microscope (Olympus IX81, Tokyo, Japan). Each micro-chamber had an individual inlet and outlet tube (Ø = 0.8 mm). Inlet tubes were placed in Corning cell culture flasks containing the desired source water (specified in the experimental design, below) and outlet tubes were connected to an 8-channel peristaltic pump (MRC, Holon, Israel), which was in turn, connected to a fraction collector (Gilson Inc. Middleton, WI, USA). The peristaltic pump ensured that flow rates (~2.6 mL h^−1^) were stable between micro-chambers and experiments, while the fraction collector allowed collection and further analysis of effluent characteristics.

### Isotopic-labelling of *V. coralliilyticus* and seawater

The YB2 *V. coralliilyticus* strain (genetically-modified to contain a plasmid-encoding for DsRed fluorescence [45]), was grown in ^15^N-labelled media (5 g L^−1 15^N 98% Celtone base powder [Cambridge Isotopes, Tewksbury, MA, USA], 2 g L^−1^ glucose, 50 μg mL^−1^ kanamycin in FSW) for 12 h in a temperature-controlled, shaking incubator (31.0 °C, 150 rpm). The bacterial suspension was centrifuged (10 min, 3500 rpm), the supernatant was decanted, and an equivalent volume of FSW was added. The sample was vortexed and returned to the incubator (31.0 °C, 0 rpm) for a further 4 h to allow non-motile *V. coralliilyticus* to sink. Motile bacteria were then collected and placed in sterile Corning cell culture flasks (Sigma Aldrich, St. Louis, MI, USA), immediately prior to the beginning of each experiment. The cultures were washed twice in FSW just before use.

Preparation of ^13^C-labelled seawater was started 3 h prior to each experiment, by addition of a defined volume of 1 M HCl (which shifted the pH to ~3 and the carbon equilibrium to >99 % dissolved CO_2_). All dissolved inorganic carbon was removed from the media by bubbling the solution with air for 2 h. The pH was readjusted to normal (pH 8) using a defined amount of 1 M NaOH. Isotopically-labelled bicarbonate (NaH^13^CO_3_, 98% atom, Sigma-Aldrich, Germany) was then added to a concentration of 2.2 mM.

### Isotope labelling experiments

#### Experiment 1. How does daytime challenge of *P. damicornis* with *V. coralliilyticus* impact ^13^C-assimilation and translocation?

A schematic of the experimental design is provided in Fig. 1b. Four *P. damicornis* fragments were placed in their micro-chambers at 03:30. At 06:30, the light provided by the microscope’s transmitted light (250 μmol photons m^−2^ s^−1^) was activated. Two fragments were challenged with the inoculum (~10^8^ cells mL^−1^ of ^15^N-labelled *V. coralliilyticus*), suspended in ^13^C-labelled FSW. The other fragments acted as non-challenged metabolic controls, and were therefore exposed solely to ^13^C-labelled FSW. The very high experimental *Vibrio* inoculation density was chosen to elicit an infection response within a reasonably short time frame [37, 39, 45] to be compatible with the conducted isotopic-labelling experiments and thus emphasizes the mechanistic point of view rather than representing an ecologically relevant scenario. Inoculation lasted 2 h, after which all fragments were supplied with pathogen-free ^13^C-labelled FSW. Images of coral fluorescence were taken at four fixed positions on the coral surface, every 10 min throughout the experiment. Fluorescence was captured in three channels, which allowed us to monitor the response of the coral host (green fluorescent protein; [GFP] Ex: 490 nm, Em: 535 ± 50 nm), *Symbiodinium* (chlorophyll; Ex: 490 nm, Em: 660 ± 50 nm), and *V. coralliilyticus* (DsRed; Ex: 555 ± 20 nm, Em: 590 ± 33 nm). This real-time approach allowed us to identify and stop the experiment at discrete stages in the infection process based on the occurrence and severity of disease symptoms, such as lesions, or biofilm formation. Once fragments developed these visible signs of infection, they were immediately fixed for subsequent analysis of cellular ultrastructure and isotopic enrichment levels (see below). Non-challenged fragments were fixed at the same time as challenged fragments, so that we could subsequently compare how *V. coralliilyticus* modified ^13^C-assimilation in *Symbiodinium* and the translocation of ^13^C-labelled photosynthates to their host using the NanoSIMS. Samples were fixed for 1 h at room temperature and then 12 h at 4 °C using a MOPS-based solution (0.5 M MOPS, 10 mM MgSO_4_, 275 mM EDTA, 2.5 M NaCl, 4% PFA, 0.1% glutaraldehyde [GA]). This experiment was repeated twice, using two different *P. damicornis* colonies (with *n* = 2 fragments *per* colony).

#### Experiment 2. How does night-time challenge of *P. damicornis* with *V. coralliilyticus* impact the metabolic turnover of ^13^C?

A schematic of the experimental design is provided in Fig. 1c. Four *P. damicornis* fragments were placed in their micro-chambers at 03:30. At 06:30, the light provided by the microscope’s transmitted light was activated (250 μmol photons m^−2^ s^−1^) and all fragments were supplied with ^13^C-labelled FSW for one light period (13.5 h: from 06:30 to 20:00). The light was then switched off and the corals supplied with non-labelled FSW. Two fragments were then immediately challenged with the inoculum (~10^8^ cells mL^-1^ of ^15^N-labelled *V. coralliilyticus*) while the other two non-challenged metabolic controls were exposed solely to non-labelled FSW. Inoculation with *Vibrio* lasted 2 h, after which all fragments were supplied with pathogen-free, non-labelled FSW. As described above, infection progression was monitored in real time and challenged and non-challenged fragments were fixed at the same time point, according to the degree of symptoms of infection. This paired design enabled us to compare how infection impacted the metabolic turnover and loss of ^13^C in *Symbiodinium* and in the host during the night phase.

At the end of both experiments, samples were rinsed (3 × 10 min) in fix-salt solution (0.5 M MOPS, 0.2 M MgSO_4_, 1 M NaCl, 0.1 M EDTA, 0.1% GA in RNase-free H_2_O) and re-suspended in 0.5 M EDTA until the skeleton was fully decalcified (~4–6 days). A final rinse (1 × 10 min) in fix-salt solution was performed before samples were re-suspended in H_2_O for transportation to the Laboratory for Biological Geochemistry (EPFL, Switzerland).

### Measurement of effluent characteristics

Throughout the experiments, effluents were collected at alternating time intervals (30 min bulk volume aliquots) to either quantify bacteria abundance or measure MMP activity in the seawater. Bacteria fractions were PFA-fixed (1%), vortexed, and 20 μL was removed, diluted in distilled water (1:10), and stained with SYBR-gold (Invitrogen). Samples were then transferred to a flow cytometer (iCyt Eclipse, Biolegend, San Diego, CA, USA) to quantify bacterial abundance by measuring the fluorescence of the SYBR-gold stain (Ex: 488 nm, Em: 525 ± 25 nm) and comparing it to a standard curve. The proportion of bacteria retained by each coral fragment during the 2 h inoculation period was calculated as the relative difference between the input inoculum density and measured output effluent density.

Effluent fractions intended for MMP analysis were passed through a 0.22 μm filter syringe (Millipore) and collected on a MMP-specific fluorogenic substrate (Calbiochem MMP-430 2/MMP-7 Substrate). The resulting filtrate was imaged using a microplate reader (Tecan Infinite M200pro) that was programmed to record fluorescence (Ex: 325 nm, Em: 393 ± 20 nm) every 90 s over a 40 min period at 31.0 °C [39].

### Sample preparation for imaging

Single polyps were micro-dissected, orientated, and embedded in 1.5% low melting agarose to ease handling of these small tissue samples and to preserve any fragile areas with tissue damage. The agarose was cut into cubes, which were placed in 1% osmium tetroxide in distilled water for 1 h. A series of washes in distilled water (4 × 10 min) and ethanol (3 × 10 min in 50, 70, 90 and 100%) followed, before the samples were embedded in Spurr’s resin. Sections (both 70 nm and 500 nm in thickness) were taken from the mouth region of the polyp using a 45° diamond knife (Diatome, Hatfield, PA, USA). The pharynx of the polyp was chosen as the area of interest, because this is where pathogens were observed to accumulate, both in this study, and in previous studies [37, 39]. The resulting thin sections (70 nm) were placed on Formvar/C–coated copper grids, for Scanning Transmission Electron microscopy (STEM), while semi-thin sections (500 nm) were mounted on round glass slides (10 mm) for NanoSIMS imaging.

### STEM imaging

Sections were counterstained in 4% uranyl acetate and Reynolds lead citrate solution (Electron Microscopy Sciences). Images were taken on a GeminiSEM 500 field emission scanning electron microscope (Carl Zeiss Microscopy GmbH, Jena, DE), at 20 kV, aperture size 20 µm, and WD 6.2 mm, using the bright field segment of a diode STEM detector (aSTEM, Zeiss). Image treatment and analysis was performed in Fiji and Adobe Photoshop. (For clarity, a knife mark was removed by blocking distinct low spatial frequencies in their corresponding Fourier image and performing an inverse Fourier transform for a reconstructed image. The original image is available upon request.)

**Fig. 2:**
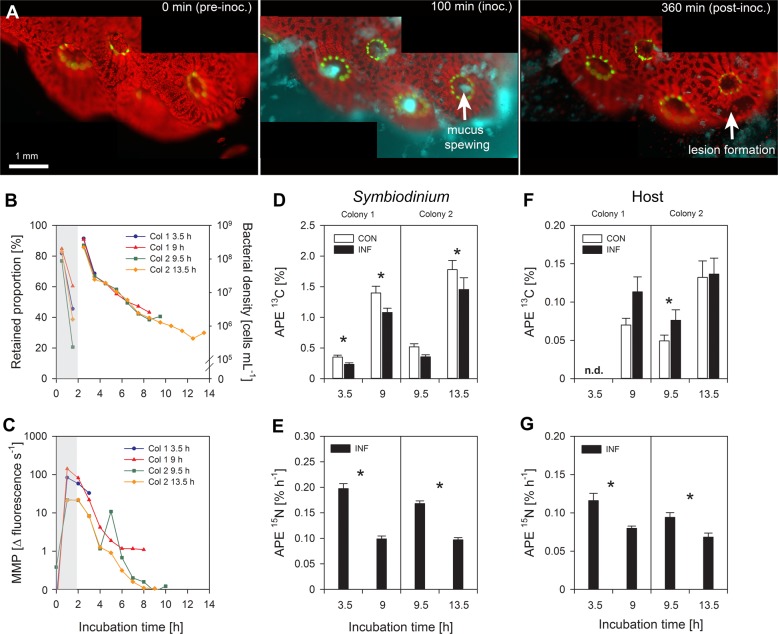
Characteristic infection of *Pocillopora damicornis* with *Vibrio coralliilyticus* in the daytime. **a** Representative images of the initial health of the coral fragment before inoculation (pre-inoc.), mucus spewing during the inoculation period (inoc.), and the subsequent formation of lesions following inoculation (post-inoc.). Colours represent: *P. damicornis*-derived green fluorescent protein (GFP; green), *Symbiodinium*-derived chlorophyll (red), and *V. coralliilyticus*-derived DsRed (cyan). **b** The proportion of bacteria retained by the fragment during inoculation (left axis), and the bacterial density in the effluent post-inoculation (right axis). **c** Matrix metalloprotease (MMP) activity measured in the effluent throughout the experiment. **d**, **f** NanoSIMS analysis of ^13^C-enrichment in (**d**) *Symbiodinium* and (**f**) host tissue in non-challenged (white) and *V. coralliilyticus*-challenged corals (black). Bars represent means ± SE. Asterisks denote significant changes (*p* < 0.05) in APE ^13^C between non-challenged and *V. coralliilyticus*-challenged corals (see Table 1 for statistical output). Note ^13^C-enrichment levels were not detectable (n.d.) in the host tissue of the fragment fixed after 3.5 h of incubation. **e**, **g** NanoSIMS analysis of the hourly ^15^N-enrichment in (**e**) *Symbiodinium* and (**g**) host tissue in *V. coralliilyticus*-challenged corals. Asterisks denote significant changes (*p* < 0.05) in hourly APE ^15^N between incubation times (see Table 2 for statistical output)

### NanoSIMS imaging

Sections were gold-coated before being transferred to the NanoSIMS. Images (either 40 × 40 or 50 × 50 µm in size) were obtained by rasterizing a 16 keV Cs^+^ primary ion beam, focused to a spot-size of about 150 nm, across the sample surface. Settings were kept constant (dwell time = 5 ms; number of pixels = 256 × 256, layers = 5) between images. The NanoSIMS instrument was tuned to a minimum mass resolving power of >8000 (Cameca definition; enough to avoid interferences in the mass-spectrum), and secondary ions: ^12^C_2_^−^, ^13^C^12^C^−^, ^12^C^14^N^−^ and ^12^C^15^N^−^ were measured in electron-multipliers. Images were taken of isotopically-unlabelled coral tissue (prepared and measured in an identical manner) at the start of each day of analysis in order to provide isotopic controls and to check instrument performance. A total of 142 images were taken, resulting in an average of 76 *Symbiodinium* cells being imaged *per* experimental sample. Data was extracted from drift-corrected images using L’IMAGE (Dr. Larry Nittler, Carnegie Institution of Washington). Regions of interest (ROIs) were drawn around individual *Symbiodinium* cells and the host gastrodermis (excluding symbionts), using the contour lines on the ^12^C^14^N^−^ image. These ROIs were then used to quantify the average enrichment of ^13^C and ^15^N in each partner. Note that the embedment of material in plastic resin dilutes the signal of carbon, and thus yields lower ^13^C levels during the subsequent NanoSIMS analysis [46]. The values we measured should thus be considered minimal estimates of carbon enrichment. To minimize the variability of this, all samples were embedded using the same batch of resin, under the same conditions. Our measured values were subsequently expressed as atom percent excess (APE, in %). This first required the conversion of the measured ^13^C^12^C^−^/^12^C^12^C^−^ or ^15^N^12^C^−^/^14^N^12^C^−^ ratios in the sample (R_sample_) and in the unlabelled sample, (R_control_) into fractions (eq. 1; where X is either the sample or the control [47]).

1 $$F_X = \frac{{R_X}}{{1 + R_X}} \times 100$$

Data were then expressed as APE (Eq. 2).

2 $${\mathrm{APE}}[{\mathrm{\% }}] = \left( {F_{\mathrm{sample}} - F_{\mathrm{control}}} \right)$$

The resulting ^13^C values were analysed as APE (%). The ^15^N values however, were normalized to incubation time [i.e. APE ^15^N, % h^−1^] prior to analysis, in order to test whether the rate of bacterial-derived enrichment was constant between fragments fixed at different time-points. Note that *Symbiodinium* cells with a diameter of less than 3 μm were excluded from the dataset because these were deemed unlikely to be representative of the response of whole cells.

### Statistical analysis

Fragments fixed at different incubation time-points were considered as independent replicates. *Symbiodinium* and host datasets were analysed separately. Non-parametric Wilcoxon signed-rank tests were used to compare ^13^C-enrichment levels (APE ^13^C, %) in *V. coralliilyticus-*challenged corals with non-challenged control corals, because the data did not fulfil the criteria required for normality. Non-parametric Wilcoxon signed-rank tests were also used to test if the hourly rate of bacterial-derived ^15^N-enrichment was constant between fragments and within colonies, fixed at different time points.

## Results

### Experiment 1. The effect of *V. coralliilyticus* challenge on ^13^C-assimilation and translocation

To determine whether *V. coralliilyticus* challenge modifies autotrophic C-assimilation in *Symbiodinium* and the coral host, coral fragments were challenged with ^15^N-labelled *V. coralliilyticus* suspended in ^13^C-labelled seawater, in the light. Their performance was compared to non-challenged fragments, which received ^13^C-labelled seawater only. Videos of both the non-challenged incubations and the *V. coralliilyticus* inoculations are provided as Supplementary Information (Videos S1-S8). No signs of physiological stress were observed in non-challenged fragments; polyps were outstretched and active throughout the experiment, and host tissue retained its confluence (Video S1-S4). Clear signs of physiological stress were observed in all coral fragments challenged with *V. coralliilyticus* (Videos S5-S8), but signs of infection only developed in three out of the four fragments (i.e. only three fragments were “symptomatic”; Videos S5-S7). Representative images are provided in Fig. 2a.

**Fig. 3:**
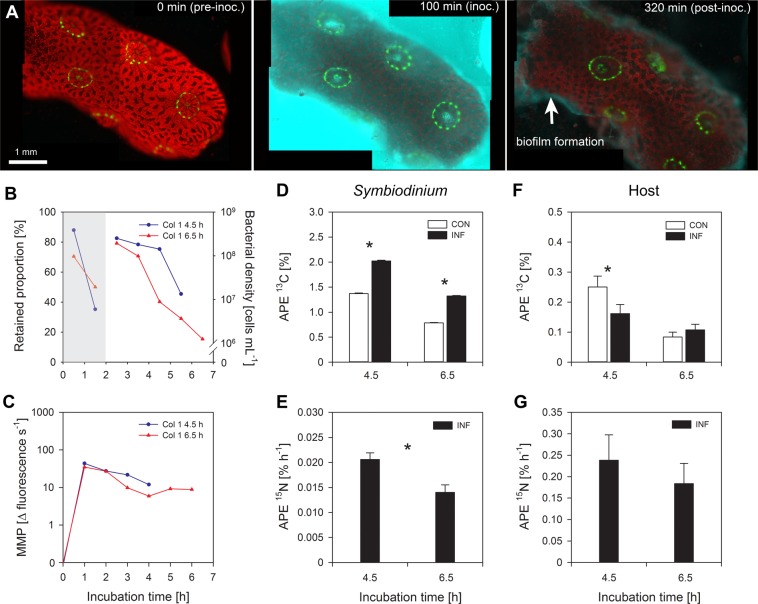
Characteristic infection of *Pocillopora damicornis* with *Vibrio coralliilyticus* at night. **a** Representative images of the initial health of the coral fragment before inoculation (pre-inoc.), the lack of mucus spewing during the inoculation period (inoc.), and the subsequent mucus film formed following inoculation (post-inoc.). Colours represent: *P. damicornis*-derived green fluorescent protein (GFP; green), *Symbiodinium*-derived chlorophyll (red), and *V. coralliilyticus*-derived DsRed (cyan). **b** The proportion of bacteria retained by the fragment during inoculation (left axis), and the bacterial density in the effluent post-inoculation (right axis). (**c**) Matrix metalloprotease (MMP) activity measured in the effluent throughout the experiment. **d**, **f** NanoSIMS analysis of the ^13^C-enrichment in (**d**) *Symbiodinium* and (**f**) host tissue in non-challenged (white) and *V. coralliilyticus*-challenged corals (black). Bars represent means ± SE. Asterisks denote significant changes (*p* < 0.05) in APE ^13^C between non-challenged and *V. coralliilyticus*-challenged corals (see Table 1 for statistical output). **e**, **g** NanoSIMS analysis of the hourly ^15^N-enrichment in (**e**) *Symbiodinium* and (**g**) host tissue in *V. coralliilyticus*-challenged corals. Asterisks denote significant changes (*p* < 0.05) in hourly APE ^15^N between incubation times (see Table 2 for statistical output)

Inoculation with bacteria caused polyp contraction for the first 20 min. After ~30 min, *V. coralliilyticus* were observed to accumulate in the polyp’s pharynx. This was followed by repeated expansion and contraction of the polyps, causing the spewing of large amounts of bacteria-laden mucus from the polyp mouth (Fig. 2a). Interestingly, the one fragment that did not develop lesions (i.e. was asymptomatic) appeared to release mucus over a longer time period (Video S8). All symptomatic fragments developed lesions in the coenosarc tissue (Fig. 2a), but timing of lesion formation differed between fragments. One developed lesions after ~200 min (fixed at 3.5 h post-inoculation [hpi]; Video S5), while two developed lesions after ~300 min (fixed at 9 and 9.5 hpi, respectively; Videos S6, S7). The asymptomatic fragment was fixed at the end of the light period (i.e. at 13.5 hpi; Video S8).

Analysis of the system effluents revealed that the number of *V. coralliilyticus* cells retained by fragments during the first 30 min of inoculation (when polyps were contracted) was similar in all fragments (~80% of the inoculum; Fig. 2b, left axis). Ninety minutes after the start of the inoculation however (after spewing had begun), there was a disparity between the fragments with regards to the number of bacteria retained within the system (which ranged from 20 to 62%; Fig. 2b, left axis). There was no difference in the amount of *V. coralliilyticus* in the effluent *post-*inoculation (i.e. *Vibrio* were washed out of all chambers at similar rates; Fig. 2b, right axis). MMP activity levels in the effluent followed a similar trend to the bacterial density data; peaking during inoculation and then declining throughout the experiment (Fig. 2c).

**Fig. 4:**
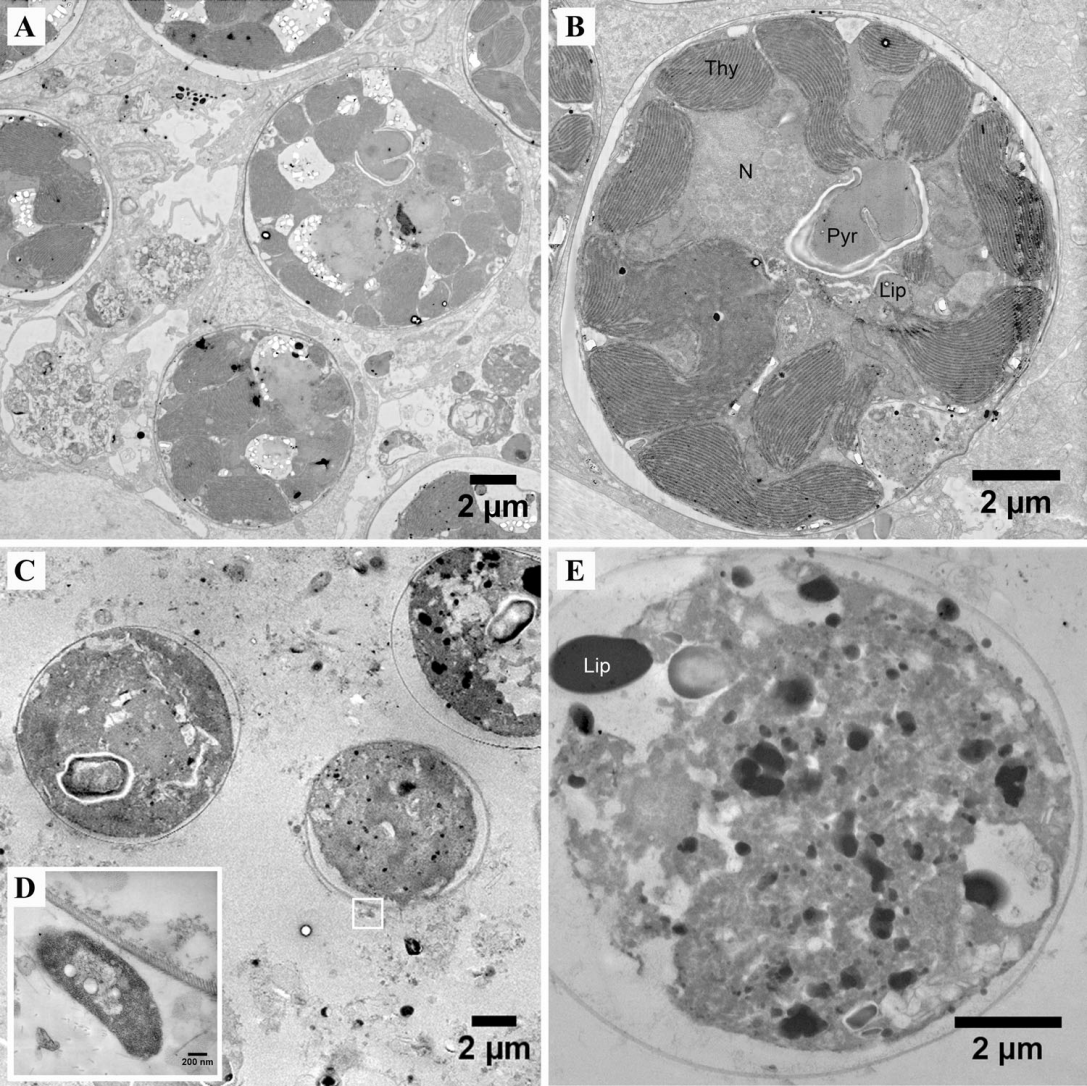
Scanning transmission electron microscopy images showing the impact of light and dark inoculations on host tissue and *Symbiodinium* ultrastructure. **a** Host tissue and **b** *Symbiodinium* cells were intact following challenge by *Vibrio coralliilyticus* in the light, but **c** host tissue was highly necrotic and contained numerous pathogens (**d**; inset) in the dark. **e** *Symbiodinium* cells were also necrotic and highly dissociated in the dark infections and lipids were observed leaking from the cell. Abbreviations: thylakoid membrane (Thy), nucleus (N), pyrenoid (Pyr), lipids (Lip). Scale bars are indicated on each image

STEM imaging of the polyps in control and infected corals revealed that host tissue was intact and bore no signs of dissociation or necrosis (Supplementary Information, Fig. S1A; Fig. 4a). *Symbiodinium* were similarly unaffected, displaying no structural abnormalities (Supplementary Information, Fig. S1B; Fig. 4b).

NanoSIMS analysis revealed that *Symbiodinium* in *V. coralliilyticus*-challenged fragments had lower APE ^13^C than those in non-challenged fragments in the two tested colonies (Fig. 2d). In three out of four fragments (those fixed after 3.5, 9 and 13.5 h) these differences were statistically significant (Table 1); and APE ^13^C was reduced by 26 ± 7% (s.d). The exception (colony 2; fixed after 9.5 h), showed a similar reduction in symbiont performance, but the difference was non-significant (Table 1) due to the high variability in symbiont performance in this fragment. Host tissue enrichment generally showed the opposite trend; corals challenged by *V. coralliilyticus* tended to have higher APE ^13^C than their control counterparts (Fig. 2f). Only one fragment showed significant differences (colony 2; fixed after 9.5 h), increasing by 57% relative to the control (Table 1). In terms of hourly APE ^15^N, both *Symbiodinium* (Fig. 2e) and host tissue (Fig. 2g) accumulated ^15^N at a slower rate in fragments that showed a later onset of disease symptoms (Table 2).

**Table 1:** Statistical output from Wilcoxon signed-rank tests, showing the effect of *Vibrio coralliilyticus*-challenge on ^13^C enrichment (APE ^13^C, %) in *Symbiodinium* and their host coral, *Pocillopora damicornis*

Inoculation conditions	Colony	Incubation time [h]	Region of interest	*χ* ^2^	*p*	Mean Δ APE ^13^C
Day [Exp. 1]	1	3.5	*Symbiodinium*	9.831	**0.0017**	−34%
			Host	–	–	–
		9	*Symbiodinium*	7.410	**0.0065**	−23%
			Host	2.939	0.0865	+61%
	2	9.5	*Symbiodinium*	0.103	0.7484	−31%
			Host	4.817	**0.0282**	+57%
		13.5	*Symbiodinium*	7.978	**0.0047**	−18%
			Host	0.125	0.7237	+5%
Night [Exp. 2]	1	4.5	*Symbiodinium*	19.530	**<** **0.0001**	+48%
			Host	3.891	**0.0486**	−35%
		6.5	*Symbiodinium*	16.592	**<** **0.0001**	+68%
			Host	0.771	0.3798	+28%

Chi-squared (*χ*^2^) and *p-*values are provided, with significant changes (*p* < 0.05) highlighted in bold. The relative difference in APE ^13^C (depicted in Fig. 2d, f and 3d, f) between non-challenged metabolic control corals and *V. coralliilyticus*-challenged corals is also provided. Note that labelling above control levels was not detectable in the host tissue of the fragment that originated from colony 1 and was fixed after 3.5 h of incubation

**Table 2:** Statistical output from Wilcoxon signed-rank tests, showing the effect of incubation time on the hourly ^15^N enrichment (APE ^15^N, % h^−1^) in *Symbiodinium* and their host coral, *Pocillopora damicornis*

Inoculation conditions	Colony	Region of interest	*χ* ^2^	*p*	Mean Δ hourly APE ^15^N
Day [Exp. 1]	1	*Symbiodinium*	52.842	**<** **0.0001**	−50%
		Host	8.308	**0.0039**	−31%
	2	*Symbiodinium*	61.140	**<** **0.0001**	−42%
		Host	6.667	**0.0098**	−27%
Night [Exp. 2]	1	*Symbiodinium*	10.368	**0.0013**	−32%
		Host	0.2135	0.6441	−23%

Chi-squared (*χ*^2^) and *p*-values are provided, with significant changes highlighted in bold. The relative difference in hourly APE ^15^N (depicted in Fig. 2e, g and 3e, g) between fragments fixed at different times is also provided

### Experiment 2. The effect of *V. coralliilyticus* challenge on ^13^C-turnover

To determine how *V. coralliilyticus* challenge modifies ^13^C-turnover in *Symbiodinium* and the coral host, fragments were photo-autotrophically labelled with ^13^C for one light period (13.5 h) and then challenged with ^15^N-labelled *V. coralliilyticus* and turnover of ^13^C monitored in non-labelled seawater in the dark. Again, their performance was compared to that of non-challenged corals, which received non-labelled seawater only. Videos of the non-challenged incubations and the *V. coralliilyticus* inoculations are provided as Supplementary Information (Videos S9-S12). No clear signs of physiological stress were observed in non-challenged fragments, polyps were extended and active throughout the experiment (Video S9, S10).

Both of the *V. coralliilyticus*-challenged fragments were clearly symptomatic (Fig. 3a; Video S11, S12), however, symptoms were markedly different from infections observed in Experiment 1, as well as from similar experiments performed in the same system [39]. Addition of *V. coralliilyticus* triggered polyp contraction, but subsequent mucus spewing was not observed. Instead, a mucus film formed on the surface of the fragments ~60 min into the inoculation period (Fig. 3a) and continued to persist there for the duration of the experiment (Fig. 3a; Videos S11, S12). In one symptomatic fragment, structures resembling nematocysts (specialised stinging organelles used in self-defence or prey capture) covered in pathogens were observed being fired after ~350 min (Video S12). In both infected fragments, the chlorophyll signal emanating from individual *Symbiodinium* cells was observed to peak and then fade quickly, producing a sort of ‘fluorescent flash’. The timing of elevation in chlorophyll fluorescence differed between individual cells, but was synchronized between regions on the surface, generating the impression of a wave traversing the surface of the coral (Videos S11, S12). Fragments were fixed at 4.5 and 6.5 hpi, respectively, for NanoSIMS analysis.

The amount of pathogens retained by the two chambers differed; the fragment that developed symptoms earlier (i.e. was fixed at 4.5 hpi) retained more *V. coralliilyticus* during (Fig. 3b, left axis) and following the inoculation period (Fig. 3b, right axis). Despite this difference, MMP activity was similar in both fragments (Fig. 3c).

**Fig. 5:**
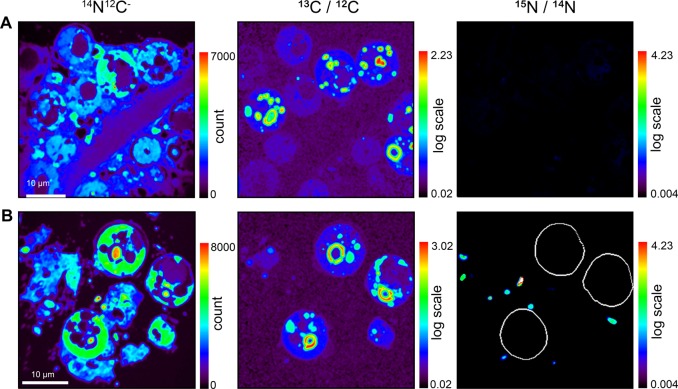
Cellular observations from inoculations conducted in the daytime and at night. Representative NanoSIMS images of (**a**) day and (**b**) night inoculations: ^14^N^12^C^−^ image showing the position of *Symbiodinium* in the host tissue; ^13^C (^13^C ^12^C/ ^12^C2) and ^15^N (^15^N/^14^N) enrichment. Colours in NanoSIMS maps represent enrichment ratios (black to red). These images permit several observations: (i) host tissue in the night-time inoculation (**b**) was severely degraded compared to tissue in the daytime inoculation (**a**) (cf. ^14^N^12^C^−^ image); (ii) *Symbiodinium* were still able to fix ^13^C-labelled seawater during inoculation in the light (δ^13^C image) just at lower levels than the non-challenged, control corals (see Fig. 1); (iii) in the daytime inoculations, very few *V. coralliilyticus* were present in the host tissue, which contrasts with the night-time inoculations, where high densities of ^15^N-labelled *V. coralliilyticus* were present; (iv) regions of interest around the *Symbiodinium* cells (white circles) show that *V. coralliilyticus* never penetrates these cells

Both STEM and NanoSIMS imaging showed there was a high density of *V. coralliilyticus* in the host tissue (Fig. 4c, d, Fig. 5). *V. coralliilyticus* was not observed inside *Symbiodinium* cells (Fig. 5). In contrast to non-challenged corals (Supplementary Information, Fig. S1C, D), both host tissue and *Symbiodinium* cells were severely necrotic (Fig. 4c, e). The cell wall and the pyrenoid were intact in some symbiont cells, but all other cellular organelles (thylakoid membranes, starch reserves, lipids) were unrecognizable (Fig. 4c, e). In some cases, lipid droplets were observed leaking from the cell (Fig. 4e). The host tissue was highly dissociated, with only small tissue clumps remaining (Fig. 4c).

The NanoSIMS analysis showed that *Symbiodinium* in *V. coralliilyticus*-challenged fragments had significantly higher APE ^13^C than symbiont cells in non-challenged fragments (Table 1; Fig. 3d). On average, the carbon enrichment of *Symbiodinium* in *V. coralliilyticus*-challenged fragments was 58% greater than in the non-challenged corals (Fig. 3d, Table 1). The pattern in the host tissue was less clear; one fragment (fixed after 4.5 h) had 35% less APE ^13^C than its non-challenged counterpart, while the other (fixed after 6.5 h) had 28% more (Table 1). The hourly APE ^15^N showed the same pattern as the light inoculation in *Symbiodinium* (Fig. 3e); cells accumulated ^15^N at a slower rate as incubation time increased (Table 2), but no difference was observed in the rate of nitrogen enrichment between different incubation times in the host tissue (Fig. 3g).

The high in situ density of *V. coralliilyticus* in the night-time inoculation, and their extremely high ^15^N content (62.1 ± 8.7 %), allowed us to identify pathogens in situ and test whether or not they obtained host-derived material (i.e. were labelled in ^13^C). Because the pathogens contained host-derived ^13^C (0.19 ± 0.11%), we tested whether there was an overall linear relationship between the enrichments in carbon and nitrogen, respectively, between pathogens and the host across time. A significant positive correlation (*ρ* *=* 0.60, *p* = 0.0029, *n* = 22) was observed between the APE ^13^C of *V. coralliilyticus* and the host (Supplementary Information, Fig. S2A), but no significant correlation (*r* = −0.26, *p* = 0.2463, *n* = 22) existed between the APE ^15^N of the bacteria and the host (Supplementary Information, Fig. S2B).

## Discussion

Pinpointing the exact mechanism involved in inducing and promoting disease in corals is complicated, because of the multi-partite nature of the coral holobiont [6] and the difference in scale of the partners. A state of disease arises if normal homoeostatic functioning is perturbed [48], which can occur as a result of modifications in host/micro-organism interactions. We partly overcame the challenge of scale differences and complex physiological responses by combining microfluidics with stable isotopes and NanoSIMS imaging. Here, we explore the links between the macro-scale behavioural response and the micro-scale nutritional response of the coral holobiont.

### Macro-scale behavioural response of the coral holobiont to bacterial challenge

The primary mechanical response of the tested fragments to a challenge with large numbers of *V. coralliilyticus* cells was similar between both the daytime and night-time infections: the coral polyps retracted into their calices in an apparent attempt to isolate themselves from the pathogens in the surrounding seawater [49]. In the first experiment, where inoculation occurred during the light period, fragments expanded their polyps shortly after the initial contraction and rhythmic retraction and expansion were observed, accompanied by spewing of bacteria-laden mucus from the pharynx region. This behaviour was then followed by changes in tissue morphology and confluence and lesions began appearing on the coral surface. It is possible that coral fragments with polyps that were more efficient at spewing mucus, developed surface lesions later. We attempted to quantify the development of lesions using image analysis, but the topography of the surface and the timing between images made it difficult to accurately distinguish between polyp movement and induced tissue damage (Supplementary Information, Fig. S3). Lesions, however, always began in the coenosarc tissue immediately surrounding the polyp, irrespective of the timing of their development. It is unclear whether the coenosarc is specifically targeted by *V. coralliilyticus* or whether the tissues in this area are simply thinner than other areas of the coral surface, and thus, more susceptible to penetration and tearing. Regardless, this finding supports the hypothesis of “polyp bail-out”, as a host-mediated defence strategy to escape an undesirable environmental condition or the onset of disease [39, 50]. In addition, the observed responses here should be viewed in the context of a high inoculum density as previous experiments have shown that V. *coralliilyticus* densities of 10^6^ or 10^7^ cells mL^−1^ take much longer to manifest a state of disease [27, 39, 41].

The observed absence of mucus spewing by polyps in the second experiment resulted in the formation of a disperse mucus film on the surface of the fragments. As a result, coral epithelia (inner and outer surfaces) are likely exposed to *Vibrio* for longer periods of time, which may explain the high *in hospite* pathogen density observed in these fragments by STEM and NanoSIMS. Observations of mucus film formation have not previously been published for *P. damicornis*-*V. coralliilyticus* interactions, but have been observed in previous infections conducted in the Coral-on-a-Chip microfluidics system (Shapiro, Gavish personal communication). The source of this phenomena cannot be attributed solely to light/dark dynamics, as some experiments conducted by Gavish et al. under dark conditions were more similar to the light infections observed here [39]. The difference may thus be attributed either to host genotype or to some uncontrolled stress incurred by the host prior to the infection which affected the observed phenotype.

Since *Vibrios* are known to develop biofilms on both inanimate and living surfaces [51, 52], it is not surprising to find them formed on the mucus surface of the corals. Considering *V. coralliilyticus* motility is unimpeded by anoxia [53], which often characterizes biofilms, we suggest *Vibrio* virulence is not weakened, and may even be enhanced, in the dark. Because circadian rhythmicity exists in many biochemical pathways and in coral behaviour [54–56] this observation would be an interesting topic for future studies to pursue.

### Micro-scale nutritional response of the coral holobiont to bacterial challenge

#### (1) ^13^C-assimilation in *Symbiodinium* and the amount of photosynthates translocated to their host

Corals challenged with *V. coralliilyticus* in the light tended to display higher levels of carbon assimilation in the host tissue, than the control (Fig. 2f). This could be linked to the behavioural response that we observed. Mucus spewing, and mucus production in general, is energetically costly [57]. A coral ‘under attack’ is thus likely to have a higher demand for energy and, hence, translocated carbon to support the extra energy-consumption triggered by this defence mechanism. Although physiological data (i.e. photosynthetic and respiratory rates) were not obtained in this study, we suggest that *Symbiodinium* in *V. coralliilyticus*-challenged corals fix carbon at a higher rate than in non-challenged corals, in order to facilitate the increase in carbon that is translocated to their host. Consistent with this, our data show that *Symbiodinium* cells in three out of the four fragments (originating from two mother colonies) that were challenged in the light, allocated less carbon to anabolic processes than *Symbiodinium* in control corals, despite remaining structurally intact (Figs. 2d, 4b). It is possible that this reflects a general reduction in *Symbiodinium* metabolism, and thus, slower cell turnover.

Another factor to consider in the interpretation of the shift in carbon partitioning between the symbiont and the host, is the sudden increase in heterotrophically-available nutrients that were provided by the inoculum. The release of phototrophic carbon from *Symbiodinium* to their host is stimulated by the amino acid pool present in the host tissue; the so-called “host-release factor” [58]. It is possible that the high bacterial load that was present in our inoculum (>10^8^ cells mL^−1^) and/or the numerous proteins secreted by *V. coralliilyticus* during the infection process [40] modified the size or quality of the amino acid pool, stimulating additional release of excess carbon to the host. Future studies could test this hypothesis by repeating our study using a non-pathogenic *Vibrio* strain, such as *V. fisheri* (as in [39]).

#### (2) Metabolic turnover of ^13^C in *Symbiodinium* and its host

The inoculations of pre-labelled corals in the dark showed that *Symbiodinium* cells in fragments of the infected colony replaced their structural carbon at a slower rate than *Symbiodinium* in the corresponding control (Fig. 3d). This is not particularly surprising, because symbiont cells in the infected corals were severely necrotic (Fig. 4e) and were thus not likely dividing or metabolically functional at the time of fixation. What happened in the host tissue however, is less clear. In one fragment (fixed at 4.5 h) host turnover was faster under the challenge of *Vibrio* than the control (Fig. 3f), but in the other (fixed at 6.5 h), there was no difference. Interpreting this data is complicated by the severe disintegration of the host tissue and the fact that labelled material (such as lipid droplets) were observed leaking from some necrotic *Symbiodinium* cells, which could have contributed to the ^13^C-signal measured in the host tissue (Fig. 4e). One important observation that arose from the dark inoculations was that the pathogens themselves are able to assimilate host-derived carbon (Supplementary Information, Fig. S2A), probably as dissolved organic material (DOM) following the dissociation of the host tissue. MMPs are thought to play a key role in this process [39, 40]. This could be proven by repeating our experiments using a *vcpA*-mutant strain of *V. coralliilyticus*, which has been genetically-modified to block MMP production [40].

#### (3) The allocation of bacterial degradation products in *Symbiodinium* and the host

A strong ^15^N signal was observed in all pathogen-challenged corals, with levels of ^15^N-assimilation being at least two-fold higher than in healthy corals exposed to high ambient levels of nitrogen [9]. The ^15^N signal encompasses both intact bacteria and their degradation products [37]. This makes it impossible to compare values in the host tissue, because of the very different amounts of bacteria observed *in hospite*. We can compare the ^15^N signal in *Symbiodinium* because intact *V. coralliilyticus* were unable to penetrate the thick cell wall that surrounds the cell (Figs. 4, 5). Therefore, any ^15^N present in *Symbiodinium* represents the products of bacterial degradation, assimilated via one of two mechanisms: either directly, as organic metabolites (e.g. amino acids) or indirectly, after the internal recycling of ammonia [59, 60]. The reduction in ^15^N-assimilation by *Symbiodinium* that we observed in the dark suggests ^15^N-fixation is coupled with photosynthesis [9] and is thus, derived predominantly from ammonia recycling. The low rates of ^15^N assimilated in the dark however, confirms that both metabolic pathways are active and used during infection.

### Controlled dual-labelling isotope experiments: lessons learned and future directions

In the present study, we show that coral/pathogen infection dynamics (speed of progression, symptoms) depends on both the behavioural response of the host organism and the intricate nutritional interactions that occur between the symbiont and its host. It is tempting to propose that the observed defence responses (increased mucus production and the active spewing of mucus) are fuelled by an increased amount of photosynthates translocated from the symbiont to the host. However, at this point our results should be viewed within the context of the limited number of colonies tested here, the high inoculum density, and the contrasting observations from previous experiments.

Future studies should combine our approach with other physiological parameters such as photosynthesis, respiration and symbiont density. Developing microfluidic chips fitted with integrated oxygen sensors is certainly a feasible target for the field of coral research in the near-future, given that such chips already exist for algae [61, 62] and cyanobacteria [63]. But, studying the response of a complex holobiont to infectious pathogens, or indeed any other physico/chemical disturbance (temperature, salinity and pH), poses a much greater challenge than single-celled organisms. This is where the combination of microfluidics and NanoSIMS stable isotopic imaging provides new opportunities. Microfluidics allows manipulation of the input source, the conditions on stage and the collection of the effluent for biochemical analysis, while NanoSIMS imaging enables us to assign functional roles to all members of the holobiont (in terms of metabolism). Together, these techniques have the potential to help answer some of the most fundamental questions standing in coral reef research: How does the coral immune system function? What is the tipping point for the transition between health and disease? And, how will the coral holobiont respond to multiple stressors in the face of climate change [64]?

It is important to highlight here, that this approach is not limited to corals. It can be applied to study broadly the biochemical signalling that occurs between bacteria and their host [65] or to investigate the complex interactions that exist between plants, mycorrhizal fungi, and bacteria [66]. In a decade where increasing emphasis is being placed on understanding the role of microbes in symbiotic systems [67], these new technical capabilities thus open exciting new frontiers in the study of host-symbiont-microbe interactions in general.

## Supplementary information

Supplementary Information
