# Supplementary material for: Vibrio coralliilyticus infection triggers a behavioural response and perturbs nutritional exchange and tissue integrity in a symbiotic coral
Source: ISME J. 2018 Dec 12;13(4):989–1003. doi: 10.1038/s41396-018-0327-2 (PMC6462045; doi:10.1038/s41396-018-0327-2)
Supplement: Supplementary file 1 — Supplementary Information [file 41396_2018_327_MOESM1_ESM.docx]

**Supplementary Information for:**

***Vibrio coralliilyticus* infection triggers a behavioural response and perturbs nutritional exchange and tissue integrity in a symbiotic coral**

E. Gibbin^1*^, A. Gavish^2^, T. Krueger^1^, E. Kramarsky-Winter^2^, O. Shapiro^3^, R. Guiet^4^, L. Jensen-Søgaard^1^, A. Vardi^2^, A. Meibom^1,5^

^1^Laboratory for Biological Geochemistry, School of Architecture, Civil and Environmental Engineering, École Polytechnique Fédérale de Lausanne (EPFL), Lausanne, Switzerland.

^2^Weizmann Institute of Science, Rehovot, Israel.

^3^Volcani Center for Agricultural Research, Rishon LeZion, Israel

^4^BioImaging and Optics Core Facility, École Polytechnique Fédérale de Lausanne (EPFL), Lausanne, Switzerland.

^5^Center for Advanced Surface Analysis, Institute of Earth Sciences, University of Lausanne, Lausanne, Switzerland

*Corresponding author: [emma.gibbin@epfl.ch](mailto:emma.gibbin@epfl.ch)

**Fig. S1. Scanning transmission electron microscopy images showing the ultrastructure of host tissue and *Symbiodinium* in non-challenged corals.** (A) Host tissue and (B) *Symbiodinium* cells in the light [experiment 1]. (C) Host tissue and (D) *Symbiodinium* cells in the dark [experiment 2]. Abbreviations: thylakoid membrane (Thy), nucleus (N).

**
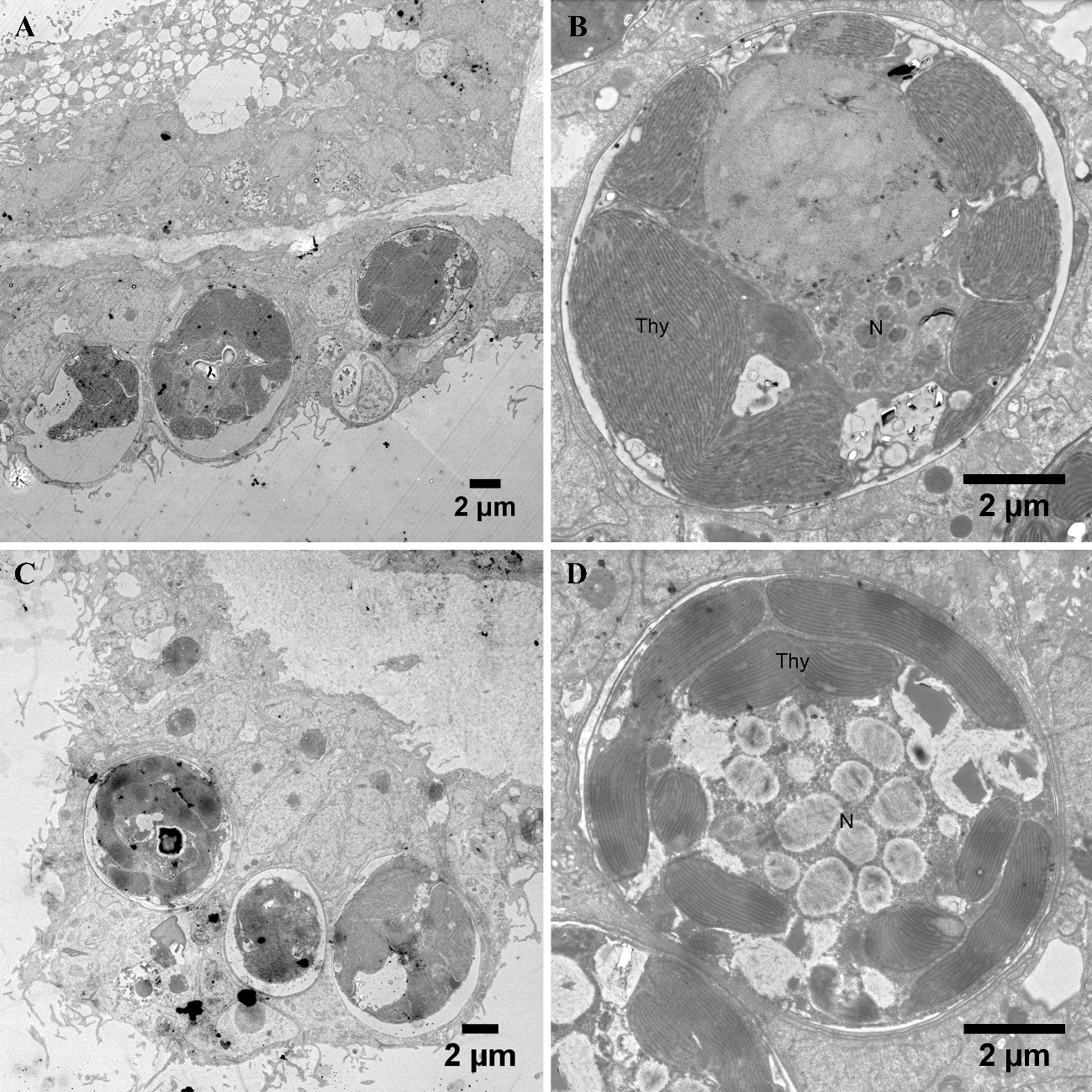
**

**Fig. S2. Transfer of metabolites from the host to the bacteria, or from bacteria to the host.** (A) Mean APE ^13^C enrichment of the pathogen relative to the APE ^13^C of the surrounding host tissue. (B) Mean APE ^15^N enrichment of the pathogen relative to the APE ^15^N of the surrounding host tissue. Correlated data points were derived from a single thresholded APE ^15^N region of interest (ROI) for all bacteria per image and the corresponding host tissue ROI (*n* = 22 images, black: 4.5h point, white: 6.5h). Overall Spearman’s rank correlation results are indicated.


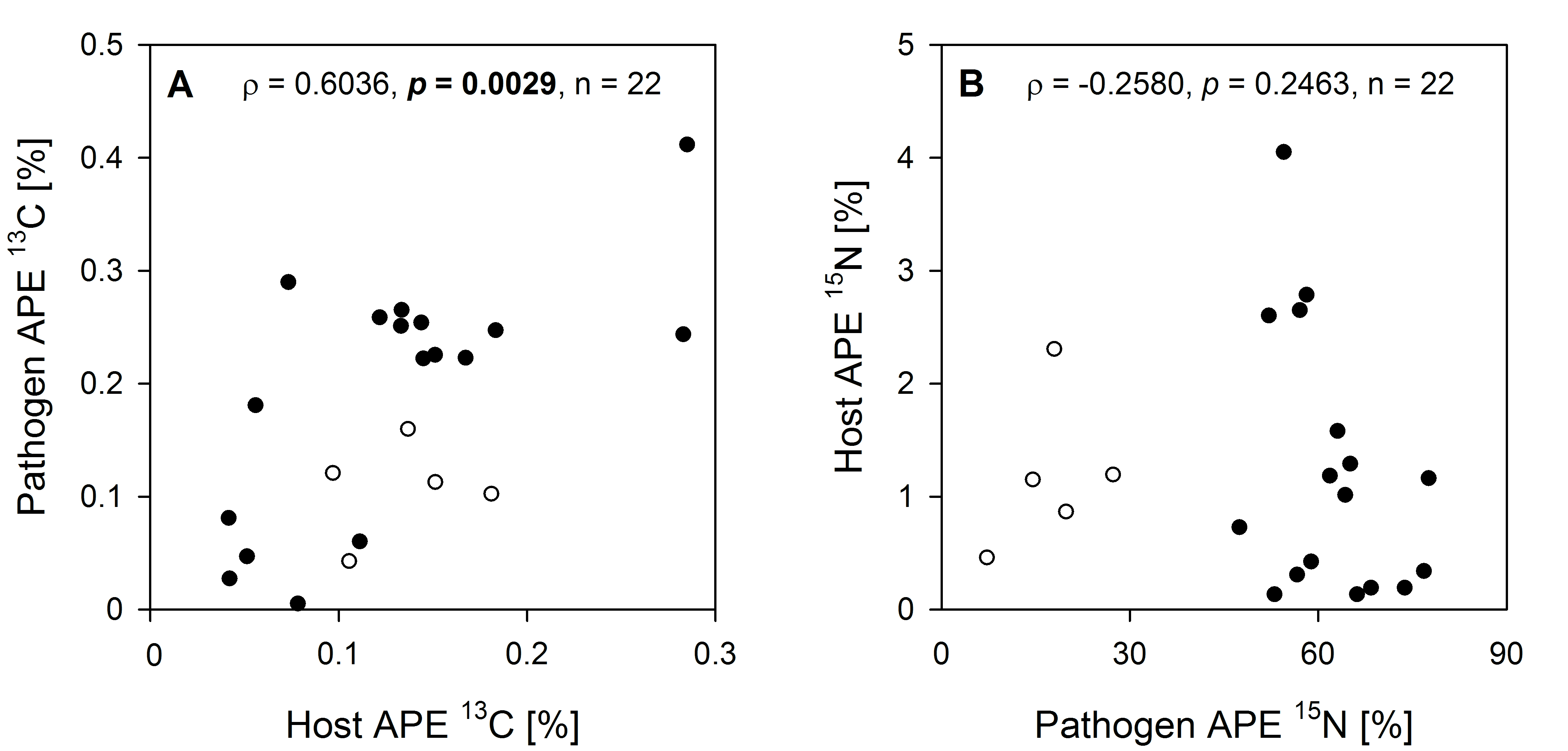


**Fig. S3. Time-integrated analysis of coral movement during light inoculations.**

The image analysis was performed in Fiji using a custom script (ImageJ macro language) to automatically analyze the time-lapse images. The script measures the area of holes in the coral tissue in order to analyze the amount of damage arising from lesions (i.e. more, or bigger holes = greater damage). First, the script homogenizes the pixel intensity by dividing each frame by a blurred version of itself (Gaussian blur, sigma = 50). Second, the script creates two masks “fill” and “holes”. For each mask, the script blurs (Gaussian blur, respectively sigmafill = 10, sigmaholes=10) and binarizes (Threshold, respectively thfill = Default, thholes= Default) the image before the binary operation “fill holes” is applied to the “fill” mask. Third, the script applies a Boolean “XOR” operator on the two masks, in order to extract the “holes”. Finally, the script measures the remaining area of the XOR mask for each frame. For visual inspection, the script generates a temporal color coded projection of the XOR mask. Depicted are *Vibrio coralliitycus*-challenged corals (left) and non-challenged, control corals (right), fixed at: (A) 3.5 h; (B) 9 h, (C) 9.5 h and (D) 13.5 h. No change in movement is black. Activity is indicated by the intensity of the colour (scaled to time).


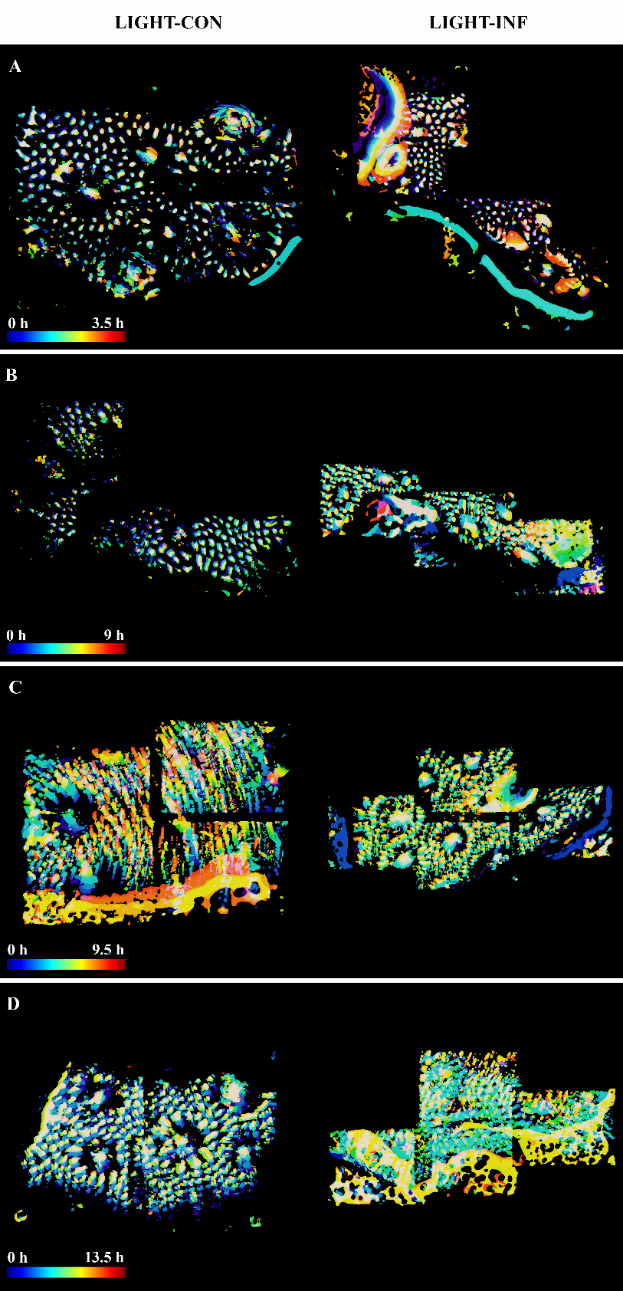


**Supplementary videos**

All videos are uploaded in MP4 quality to the Open Access repository, Vimeo (<https://vimeo.com/home>). Colours show: *P. damicornis*-derived green fluorescent protein (GFP; green), *Symbiodinium*-derived chlorophyll fluorescence (red) and *V. coralliilyticus*-derived DsRed fluorescence (cyan). Incubation information and links provided below.

**Video S1. Non-challenged *Pocillopora damicornis* fixed after 3.5 hours.** Incubation performed at 31°C, under 250 µmol photons m^-2^ s^-1^ of white light.

<https://vimeo.com/283886920>

**Video S2. Non-challenged *Pocillopora damicornis* fixed after 9 hours.** Incubation performed at 31°C, under 250 µmol photons m^-2^ s^-1^ of white light.

<https://vimeo.com/283891170>

**Video S3. Non-challenged *Pocillopora damicornis* fixed after 9 hours.** Incubation performed at 31°C, under 250 µmol photons m^-2^ s^-1^ of white light.

<https://vimeo.com/283891796>

**Video S4. Non-challenged *Pocillopora damicornis* fixed after 13.5 hours.** Incubation performed at 31°C, under 250 µmol photons m^-2^ s^-1^ of white light.

<https://vimeo.com/283892165>

**Video S5. *Pocillopora damicornis* challenged with *Vibrio coralliilyticus* and fixed at 3.5 hours post-inoculation.** Inoculation and subsequent incubation performed at 31°C, under 250 µmol photons m^-2^ s^-1^ of white light.

https://vimeo.com/270068267

**Video S6. *Pocillopora damicornis* challenged with *Vibrio coralliilyticus* and fixed at 9 hours post-inoculation.** Inoculation and subsequent incubation performed at 31°C, under 250 µmol photons m^-2^ s^-1^ of white light.

<https://vimeo.com/270069780>

**Video S7. *Pocillopora damicornis* challenged with *Vibrio coralliilyticus* and fixed at 9.5 hours post-inoculation.** Inoculation and subsequent incubation performed at 31°C, under 250 µmol photons m^-2^ s^-1^ of white light.

<https://vimeo.com/270070159>

**Video S8. *Pocillopora damicornis* challenged with *Vibrio coralliilyticus* and fixed at 13.5 hours post-inoculation.** Inoculation and subsequent incubation performed at 31°C, under 250 µmol photons m^-2^ s^-1^ of white light.

<https://vimeo.com/270070178>

**Video S9. Non-challenged *Pocillopora damicornis* fixed after 4.5 hours.** Incubation performed at 31°C in the dark.

<https://vimeo.com/283894343>

**Video S10. Non-challenged *Pocillopora damicornis* fixed after 6.5 hours.** Incubation performed at 31°C in the dark.

<https://vimeo.com/283894860>

**Video S11. *Pocillopora damicornis* challenged with *Vibrio coralliilyticus* and fixed at 4.5 hours post-inoculation.** Inoculation and subsequent incubation performed at 31°C in the dark.

<https://vimeo.com/270070207>

**Video S12. *Pocillopora damicornis* challenged with *Vibrio coralliilyticus* and fixed at 6.5 hours post-inoculation.** Inoculation and subsequent incubation performed at 31°C in the dark.

<https://vimeo.com/270070214>
